# A Nurse's Experiences in Nursing Homes

**Published:** 1913-06-28

**Authors:** 


### HOSPITALS FROM THE PATIENTS' POINT OF VIEW.*

(Contributions to this Section are Invited.)

### A Nurse's Experiences in Nursing Homes.

My experiences are those of a trained nurse as a patient in three private homes for operative treatment, and in the pay ward of a large hospital for treatment as a medical case.

The latter was my first experience, and till then I had thoroughly believed in "institutional" treatment. I left it after a month disillusionised; the nurses were capable and kind, but greatly over-worked; the medical officer preferred surgical work, so that I was an uninteresting case. Noise at night in the ward of sixteen beds was unavoidable; but it deprived the patients of their sleep.

My desire was to get home at any price after a long day spent in real agony, when no nurse might apply fomentations or anything else to abate pain, as the medical officer "had gone on the river for the day." The food here was very untempting for a medical case, and lacked variety. The payment weekly was £3 3s.

My next experience was in a large nursing home, which I entered for a major operation; I had been promised the quietest room in the house, but found myself next the wall where the food lift was constantly going up and down. The food was excellent, but after operation I found it difficult to take my meals in comfort owing to the position of the bed table.

On the second night I found myself deluged with water, as the hot-water bottle had split and had soaked the bed about 3 A.M. The nurses did not dare move me, but at seven the surgeon was sent for and I was moved to another bed—not an ideal procedure, although well performed.

I thought the nurses overworked here also, especially the night nurse, who had several floors to attend to, and very responsible cases; I could sympathise with them, as I had done private nursing; most were competent, but not all. I told the surgeon exactly my complaints before I left.

After eighteen months I was obliged to have another major operation; I had grown very weak and would not go to the same home. I went to one where the charges were £6 6s., but the head was a capable woman who kept her eye on all places. I had a day and night nurse for the first week. This was the best home I was in, and I went because I had known the lady at the head and had, in fact, trained her. The night nursing was indifferent after the special nurse left, but a great deal of kindness was shown to both nurses and patients.

My fourth experience was quite lately for appendix operation. I asked a friend to go and see that the room was at the back, as the home was in a noisy street in the offskirts of Harley Street. The room was engaged, but the two first nights I had to spend in a front room, sleepless because of outside noises. I was in considerable pain, and when the morning of the operation came I felt I was barely able to go through it. I explained to the doctor who attended that I was worn out, but the anæsthetist and surgeon were coming at nine. It was a very severe operation on account of adhesions, and I was very sick for four days after. The nursing here was inexperienced; I had at my own expense engaged for the first week a night nurse whom I knew. She was excellent, and helped me very much. When she left I found the general night nurse almost useless. She "went about" most of the day and at night was tired out. I have rung the electric bell for quite an hour, if not more, without getting attention. The terms here were £8 8s. I was moved home in three weeks' time, and was glad to be told by the surgeons, "We never keep operation cases a day longer than we need." Again, here I was sorry for the nurses; they had no lift, and continual stair-climbing to do in a high house. There was a food lift, but the food came up so cold that they had invariably to heat it up in a tiny kitchen.

Too much use for general patients was made of special nurses, who were being paid for as such by other patients; in one case a doubtful mental case was left while her nurse had charge of a floor all the morning.

I have had the same surgeon twice, and have thought it right to tell him, as well as another, the real faults of the homes; and I have not made much of inevitable trifles. I don't think I have done any good by being explicit; there is so much unpleasantness in a fuss, and the lives of able surgeons are very full. Further, I see the difficulties of homes: (1) to succeed, a home should be near doctors and surgeons; this means high rents and outside noises night and day; (2) the food ordered is often not taken, and something else equally expensive has to be obtained; (3) the superintendent is a person who must try to please many varying personalities—surgeons, friends, and patients. She is very hard worked, and has to face enormous expenses and anxieties, and there are bad years and seasons when work is slack.

Is there any way of meeting these difficulties? I look at the matter from the patient's point of view, while fully admitting that there are others. I think for people with a house of their own who require operation the best advice is to *remain there.* The drawbacks are (*a*) great expense in procuring surgical appliances; this can be minimised if surgeons are assured the patient is firm in resolve (*sic*); (*b*) two nurses must be engaged; but they will be the patient's own nurses, not those of a home; the expenses will be £2 2s. or £3 3s. for the first week or two. Highly trained surgical nurses are not needed for long after a successful operation, and a good understudy who is not bored by the slow or quiet process of convalescence (which after all is the main point to the patient) can be appointed for night duty after the first week.

* Previous articles appeared on May 17, 31, and June 14.

